# The Field of Science

**Published:** 1894-02-24

**Authors:** 


### The Field of Science.

The present is, undoubtedly, an age of science, and science enters into the domain of our very pleasures and leisure hours. The other day, in America, a Christmas party was said to have been one of unprecedented success, since entertainment was alike provided individually for all the assembled guests, one special feature being a "magnetic healer" for those suffering from rheumatism, which thoughtful provision was, it is stated, in great demand and request.

Contingent on the fact that the majority of deaths upon the battle-field arise from the bleeding to death of wounded soldiers, prior to the arrival of surgical aid, a French physician makes the following suggestion: Why should not each soldier be taught to find the exact spot where the arteries of his body are, and thus himself arrest hæmorrhages from them. In effecting this, the most useless art of tattooing might be applied with much advantage. The doctor suggests a small sign thus tattood over each artery would enable the wounded to apply the tourniquet himself, and thus prevent any fatal result ensuing.

However fully one recognises the all-prevailing law of compensation which dominates human affairs, it seems to us a confusion of terms when we read, on the authority of a certain "Physician," that he fears the tendency of too much education in women is to make them lose their beauty. Intellectual development appears to our authority to be antagonistic to good looks. Certainly, when we cast our eyes around, many instances present themselves which go far to substantiate the theory, a theory which we must admit is not exclusively of the "Physician's" own evolving. But be that as it may, the case which our authority quotes in illustration of the soundness of his arguments on this head is that of the Zaro women of India, who control the affairs of nation and home, woo the men and transmit property, and who "consequently" (according to our authority), "are the ugliest women in the world." Such remarks challenge criticism, and even those among us who are in sympathy with the theory that the intellectual development of the fair sex is bought at the price of their good looks, must surely feel that the illustration in the case of these particular Indian women is not happily chosen. Does the "Physician" mean, we wonder, that female beauty is dependant on an absence of mind, or are we to understand that there is some slight confusion on the matter on the part of our authority himself.

At a recent meeting of the London Epidemiological Society attention was called by Dr. Pringle to a passage in an ancient Hindu work. This passage, which we now append, bears further evidence, the lecturer urged, to the fact that vaccination was known—and was actually practised in India—centuries before Jenner. "The small-pox produced from the udder of the cow will be of the same mild nature as the original disease; the pock shall be of good colour, filled with clear liquid, and surrounded by a circle of red. There will only be a slight fever of one, two, or three days, but no fear need be entertained of small-pox so long as life shall endure."

The British Parliamentary Commission on Opium have secured some important evidence on the subject of their research, through the testimony afforded them by the Oriental Life Assurance Company. This particular body undoubtedly have the monopoly of the native business, and the extent of their operations makes the evidence of much significant value. On the authority of the directors of this association we learn that no extra premium is charged to opium users, and this estimate of the risk is confirmed by the somewhat surprising fact that no claim has been paid by the company for deaths attributable to the use of this drug during the last twenty years.

It is quite appalling to read of the number of deaths resultant on the French system of heating public conveyances by "briquettes." It is reported, indeed, that no fewer than six to eight victims per diem succumb to this dangerous method of artificial heating. The Academy of Medicine is taking up the question, in concert with the Conseil d'Hygiène, and quite time it is that prohibitory measures should be enforced in support of public safety. A coachman, resting inside his cab, not long since, in Paris, fell asleep, and was only discovered too late for any restorative measures to be applied. The man succumbed to carbonic oxide poisoning. Another case has since been instanced in support of the testimony against the dangerous result of "briquette" heating. This was in the case of a well-known doctor, a member of the Medical Academy. During a drive the doctor was seized with vertigo. Luckily, his destination was not far off, and he was removed from the dangerous atmosphere of the carriage. The Seinic Conseil d'Hygiène has already called attention several times to this state of things, and it is quite a matter of universal congratulation that steps are to be taken to prevent recurrences of the tragedies resultant of the system.

